# Ravens attribute visual access to unseen competitors

**DOI:** 10.1038/ncomms10506

**Published:** 2016-02-02

**Authors:** Thomas Bugnyar, Stephan A. Reber, Cameron Buckner

**Affiliations:** 1 Department of Cognitive Biology, University of Vienna, Althanstrasse 14, 1090 Wien, Austria; 2 Haidlhof Research Station, University of Vienna and University of Veterinary Medicine, 2540 Bad Vöslau, Vienna; 3 Department of Philosophy, University of Houston, 4300 Calhoun Road, Houston, Texas 77004, USA

## Abstract

Recent studies purported to demonstrate that chimpanzees, monkeys and corvids possess a basic Theory of Mind, the ability to attribute mental states like seeing to others. However, these studies remain controversial because they share a common confound: the conspecific's line of gaze, which could serve as an associative cue. Here, we show that ravens *Corvus corax* take into account the visual access of others, even when they cannot see a conspecific. Specifically, we find that ravens guard their caches against discovery in response to the sounds of conspecifics when a peephole is open but not when it is closed. Our results suggest that ravens can generalize from their own perceptual experience to infer the possibility of being seen. These findings confirm and unite previous work, providing strong evidence that ravens are more than mere behaviour-readers.

## Abstract (web summary)

Theory of Mind experiments in animals have not previously discounted the possibility that individuals follow their competitors′ behavioural cues. Here, Bugnyar *et al.* show that ravens consider the possibility that they are being watched when caching food, even when they cannot see a conspecific competitor.

## Graphical abstract

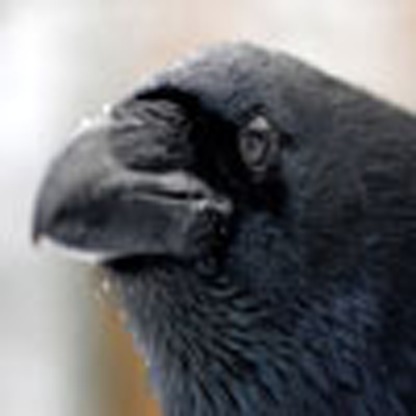

Knowing what others see would provide animals with an advantage when competing for food, for it would allow them to predict which items might become the subject of disputes. In line with this ecologically based assumption, behavioural experiments manipulating the visibility of food in face-to-face competition tasks have revealed that non-human primates^1^,^2^,^3^ and corvids^4^,^5^,^6^ react to what conspecifics can and cannot see. Many researchers have thus concluded that these animals possess an understanding of perception-goal psychology—a basic ‘Theory of Mind'. However, because in most of these experiments successful performance can be achieved by tracking correlations between head cues and a competitor's behaviour, skeptics have concluded that all these experiments suffer from a ‘logical problem' that renders them unable to empirically distinguish representations of directly observable cues from a genuine representation of ‘seeing'^7^,^8^,^9^,^10^.

In an attempt to overcome this problem, researchers have tried to develop designs that control for others' gaze. In a landmark study, Emery and Clayton^4^ showed that scrub-jays recache food after being watched at caching. The design controlled for occurrent gaze cues in the test by blocking visual access to the competitor during recaching. However, it did not control for a memory of past gaze cues, as the competitor was present during the initial caching episode—and so even this elegant design can be explained by appeal to representations of previously observed gaze cues alone^7^,^8^. Recently, Schmelz *et al.*^11^,^12^ showed that chimpanzees predict others' preferences in a back-and-forth foraging game even when they never saw their competitors' gaze at the stimuli used in the test, and Ostojić *et al.*^13^,^14^ found that Eurasian jays can predict the food preferences of their mates only if they see what they have been prefed, even if denied prior visual access to the partners' response to prefeeding in this context. Although these lines of research both control for behavioural cues in sophisticated ways, they focus on attributions of preferences rather than sight. Thus, it still remains an open question whether any nonhuman animal can attribute the concept ‘seeing' without relying on behavioural cues.

Like scrub-jays^15^, ravens also cache food items spontaneously and they are highly sensitive to the presence of conspecifics that may pilfer caches^16^. In particular, ravens decrease the likelihood of revealing cache locations to competitors by (i) reducing the time to finish caches, (ii) using obstacles as visual barriers for caching outside the view of competitors, (iii) delaying caching until competitors have left and (iv) staying away from already made caches as long as competitors are around^17^,^18^. Across all these studies, however, another raven was visible during the test, again raising the skeptical refrain that ravens might represent only gaze cues^10^. Suggestively, however, Stulp *et al.*^19^ and Shaw and Clayton^20^,^21^ have recently shown that jays can infer the presence of unseen conspecifics on the basis of auditory cues. These findings point the way towards the experimental design reported here, which aims to finally overcome the line-of-gaze interpretation by controlling for both (i) gaze cues in the test, by relying only on sounds to indicate the presence of a possible competitor and (ii) memories of gaze cues previously observed in the training, by denying the subjects prior access to any competitor's gaze in contexts resembling the test. Specifically, we ask if ravens can transfer knowledge from their own experience in a novel context—using peepholes to look into an adjacent room—to a caching situation in which they can hear but not see a conspecific in that room.

The experiment was conducted in two rooms separated by a wooden wall. The wall had two functional windows that allowed visual access from one room into the other. Both windows could be closed with covers. Furthermore, each window had a peephole drilled into its cover, which could be independently opened or closed (Supplementary Fig. 1). At the onset of the study, all ravens were familiar with experimenters opening or closing the windows between the rooms but were naïve about the peepholes. In an initial, baseline step, we determined the caching behaviour of individual ravens when they received food in one room and the windows to the adjacent room containing conspecifics were open (observed condition) or closed (non-observed condition; Supplementary Tables 2 and 3). Crucially, in both of these conditions, the peepholes were closed. In the next, familiarization step, each raven was individually trained to use one of the peepholes to observe and recover human-made caches in the adjacent room. In the final stage, each raven was confronted with the two baseline conditions (windows opened or closed) and an interspersed test condition, in which the windows between the two rooms were closed, but one of the two peepholes was open (Fig. 1). Rather than another raven being present in the test, however, the observation room was empty, and a hidden loudspeaker was used to play a series of sounds that were recorded from a competitor raven in the observation chamber during the *non*-observed condition.

The experimental hypothesis that ravens learn in the familiarization trials that peepholes afford seeing predicts that ravens will behave in the test as though they are potentially being observed through the peephole. Specifically, from the findings in the observed and non-observed conditions of the initial baseline step, we would expect ravens to (i) finish caches more quickly in the peephole condition than in the non-observed condition and (ii) return to improve their caches less often in the peephole condition than in the non-observed condition, and that their caching behaviour in the peephole condition would not differ significantly from their caching behaviour in the observed condition.

We show that ravens treat the test condition like the observed condition, indicating that they can generalize from their own experience using the peephole as a pilferer and predict that audible competitors could potentially see their caches. Consequently, we argue that they represent ‘seeing' in a way that cannot be reduced to the tracking of gaze cues.

## Results

### Caching with peephole as though observed

**Figure 2:**
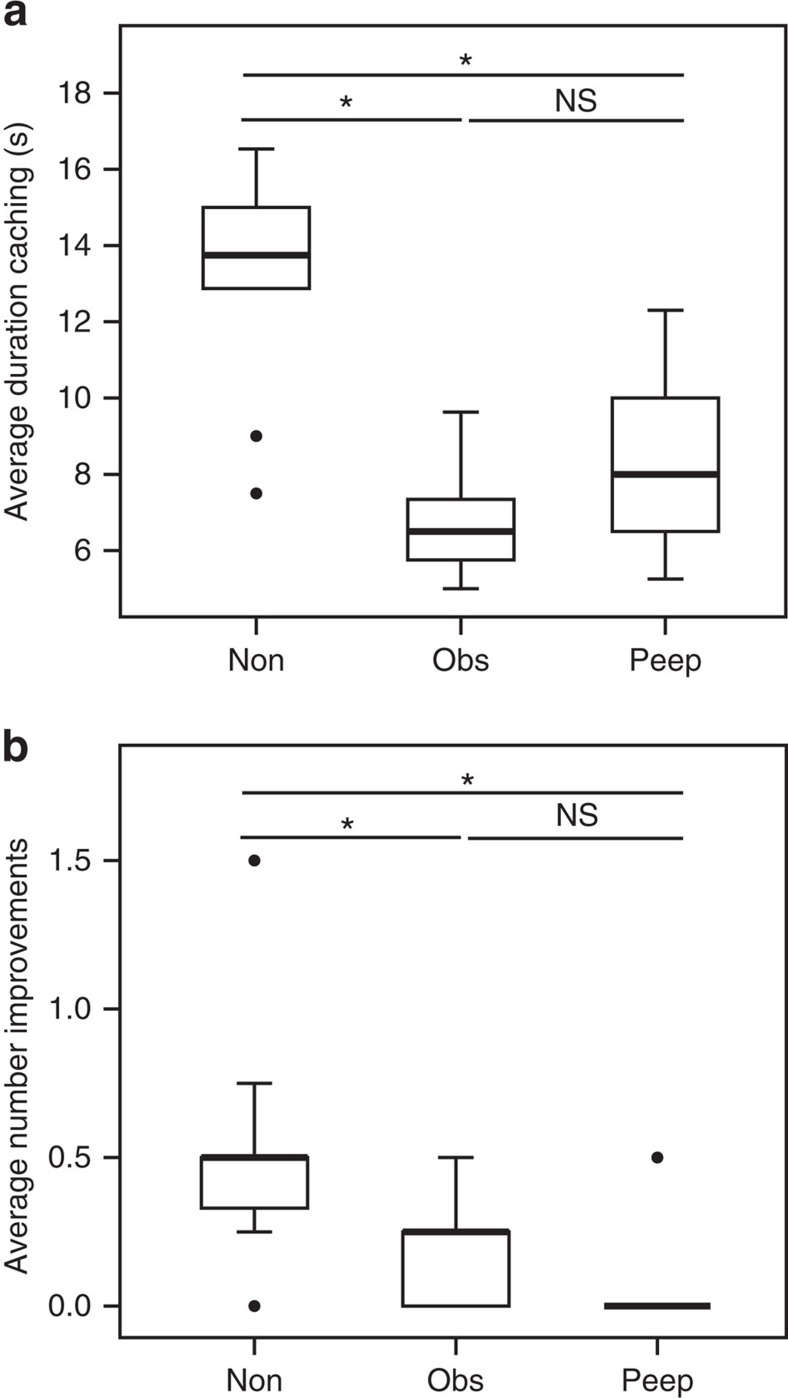
Effects of condition on caching behaviour. (**a**) Mean time to finish a cache and (**b**) mean number of revisits with improvements, in the non-observed condition (Non, total of 4 trials per 10 ravens), observed condition (Obs, total of 4 trials per 10 ravens) and peephole condition (Peep, total of 2 trials per 9 ravens). Box plots represent 25th and 75th percentiles, centre line indicates the median, whiskers represent non-outlier range and dots are outliers (Friedman test, *post hoc* Wilcoxon signed ranks test; *P<0.05; NS=non-significant).

Figure 2 summarizes the main findings. As expected, ravens differed between conditions in the time to finish a cache (*χ*^2^=14.889, d.f.=2, *P*<0.001) and the number of revisits to improve a cache (*χ*^2^=12.929, d.f.=2, *P*<0.001). These data clearly support both experimental hypotheses. The subjects finished their caches more quickly and they returned to improve their caches less often in the peephole condition than in the non-observed condition (Wilcoxon signed-ranks test, Bonferroni corrected: time to finish: *n*=9, *Z*=−2.666, *P*=0.012; revisit and improve: *n*=9, *Z*=2.539, *P*=0.024). No significant difference could be found between the peephole and the observed condition regarding these hypotheses (time to finish: *n*=9, *Z*=−2.192, *P*=0.08; revisit and improve: *n*=9, *Z*=−1.414, *P*=0.94). Note that ravens did go back to visually inspect their caches in several conditions (Friedman test: *χ*^2^=1.032, d.f.=2, *P*=0.625), indicating that they were not generally inhibited in returning to their caches in the observed and peephole conditions. This asymmetry makes adaptive sense, as prior studies have shown that observer ravens do not use gaze direction to find hidden food (despite being skilled at following others' gaze into distance and around barriers) but are very prone to show enhancement when they see someone touching an item^22^. Because all available behaviour-reading cues have been controlled for in the test condition—there is no actual competitor whose gaze could be read, and the situation is novel from the subject's perspective—these data provide clear evidence that raven social cognition cannot be reduced to behaviour-reading.

### Using peephole's limited viewing angles to hide caches

The experimental setup also allowed us to investigate whether ravens understand more detailed information about the point of view of possible observers. As there were few physical structures in the caching room that could serve as visual barriers and observers could freely move between the two windows, it was difficult for the ravens to place caches outside of view of conspecifics in the observed condition. In the peephole trials, however, our design of opening one peephole at a time enabled us to use parts of the front wall as a visual barrier. Hence, this design allowed us to test whether ravens could predict which side of the room could not be seen by a potential competitor from a particular peephole. If they could, they should avoid caching in the area in front of the open peephole only. Although particular individuals did place their caches to the left when the right hole was open and to the right when the left hole was open (see Supplementary Fig. 2 for details), the ravens as a group did not show a significant preference for caching away from the open peephole (1.78 caches made inside view versus 2.78 caches made outside view, Wilcoxon signed ranks test: *n*=9, *Z*=1.185, *P*=0.297). These results indicate that ravens need visual feedback for effectively using barriers and point towards possible limitations of their attribution skills.

## Discussion

Do the current data provide evidence that ravens have a Theory of Mind? A difficulty is finding an empirical criterion for assessing the presence of Theory of Mind that applies to the current debate over attributions of seeing in nonlinguistic animals. The most popular criterion for assessing Theory of Mind in general has been the false belief task, suggested in response to Premack and Woodruff's original article on Theory of Mind^23^. Following Wimmer and Perner's application of this test to children^24^, it has become the standard benchmark for Theory of Mind development in humans^25^. However, this criterion is not well-suited to arbitrate the current dispute, because the clearest versions of the task rely on language and because it assesses the attribution of more advanced epistemic states (like belief) rather than perceptual states (like seeing). Indeed, most of the researchers cited in the introduction concede that there is little evidence that nonhuman animals possess the full-blown metarepresentational capacities required to attribute false beliefs, but still think there is an interesting empirical debate to be had regarding whether any other social animals have the perceptual precursors to these abilities (such as the ‘secondary representations' posited in Perner's developmental model—see ref. 26).

The closest one finds to an ecumenical proposal that could arbitrate this dispute is Whiten's^27^,^28^‘intervening variable' solution (see also ref. 7 for evidence that some skeptics accept this benchmark). The current data provide evidence that ravens satisfy this criterion. The core idea of Whiten's proposal is that an animal represents an underlying mental state when it groups a variety of causes and effects together under a common internal code. As evidence for such a code, the ability to behave appropriately in novel conditions by flexibly integrating information from perceptually dissimilar situations is crucial. Our peephole test condition is novel in this sense, and the data concerning cache duration and number of improvements are consistent with an intervening variable interpretation.

Because conspecifics were never present in the peephole context in either the pretraining or the test conditions, these data demonstrate a flexible ability to individually modulate distinct behaviours to specific and novel circumstances, which cannot be reduced to a tracking of gaze cues. Recent critical discussions of the intervening variable solution have emphasized just these forms of flexibility and integration, suggesting that the explanatory power of a Theory of Mind hypothesis may lie in the capacity of the animal to learn new observable cues (such as open peepholes) to indicate the presence of a competitor's mental state^9^,^10^. The observed, non-observed and peephole conditions are clearly perceptually dissimilar, and cache speed and inhibition of revisits reflect skills that are clearly distinct.

Moreover, no combination of individual stimulus-response links can explain the full pattern of observed data. The presence of peepholes alone cannot explain the results, because the subjects lack a specific associative history for caching in the presence of peepholes; as evidence that they did not simply regard them as a familiar form of visual occlusion, note that they were initially fearful of the peepholes in familiarization trials, and one raven never achieved criterion performance. Neither can the presence of the playback sounds alone explain the results, because if the ravens could detect the difference between live and playback stimuli, the latter should be perceived as strange and elicit cautious behaviour in playback trials in general. However, the ravens did not show any inhibition in caching or returning to their caches in the test condition; they only inhibited behaviours indicative of ‘being observed', like the touching of already made caches. This selectivity in response cannot be explained by perceiving playback as a novel stimulus that elicits caution. Finally, we should note that the current results cannot be explained merely on the basis of stress or anxiety as suggested by a computational model offered recently by Van der Vaart *et al.*^29^. The stress hypothesis predicts increased caching when observed and increased revisits, but the opposite was found in our study (see also refs 30, 31).

Skeptics might further worry that the effects observed in this study were artefacts of human enculturation, given that the ravens were human-reared and had experience interacting with humans in behavioural experiments (as has sometimes been suggested to explain the social cognition of dogs and apes—see ref. 32). In short, the ravens might only have learned to use the peepholes because they have acquired special learning abilities because of the control that humans wield over their food supply. Even if this were the case, it would not constitute a good argument against the interpretation of the data offered here—unless we were prepared to accept that Theory of Mind is an artefact in humans as well. From birth, children are also extraordinarily dependent upon their caregivers for food, and are also entrained, implicitly and explicitly, to attune to the social behaviours of their conspecifics. It is crucial in cross-species comparisons that we apply the same yardstick to humans and animals^33^. Thus, although it would still be of considerable evolutionary interest to determine whether parent-raised ravens could pass the peephole test without human enculturation, this worry should not lead us to reject the current interpretation of the results.

It may also be promising to consider the present findings in terms of the ‘minimal' (as opposed to ‘full-blown') Theory of Mind recently articulated by ref. 34. Agents with minimal Theory of Mind adaptively respond to mental states by representing ‘encounterings', defined as relations between agents and objects in their visual field. Such animals can learn that ‘having encountered food (is a) a condition for performing goal-directed actions targeting that food', and can thus, to prevent theft, be motivated to prevent others from encountering their caches. To accommodate our data, this principle must be elaborated to include the computation of possible encounterings by agents even when no competitor is visible and when generalized from their own perceptual experience. This may appear to approach some of the most sophisticated criteria proposed for perceptual Theory of Mind, such as Heyes' ‘projection' test^10^. However, at least as a group the ravens' ability fell short of ‘full-blown' human Theory of Mind, as it did not enable them to anticipate the limited viewing angle from one peephole or another—although this is a sophisticated inference and it is unknown when such ability emerges in human development and the degree to which adult human subjects would demonstrate it.

In conclusion, the current experiment, together with the other recent studies on chimpanzees^11^,^12^, provides strong evidence against the skeptical hypothesis that the social cognition of nonhuman animals is limited to behaviour-reading. Peephole designs can allow researchers to overcome the confound of gaze cues, but further experimental work is needed to determine the specific limits of ravens and other animals—including humans—on such tasks.

## Methods

### Subjects and housing

Subjects were ten subadult ravens (6 males and 4 females), all hand-raised and socially housed at the Haidlhof Research Station, Austria (see Supplementary Table 1 and Supplementary Fig. 1a for details). Birds were taken as nestlings at the age of ∼4 weeks, individually marked with coloured leg bands and hand-reared to fledging by human caretakers for the purpose of behavioural studies. As fledging, they have been subjected to daily observations and cognitive tests; accordingly, they have been trained to come by name and individually participate in physical and visual isolation from their group members. All birds were fully habituated to the experimental setup and testing procedure before the onset of the study but none of them had any experience with caching experiments.

The experiment was conducted from May to October 2013. At the time of testing, nine birds were in the second year of life and one bird (female Astrid) was in its fourth year. All birds but this older female were kept in one social group in the non-breeder area (Supplementary Fig. 1a); the older female had already formed a pair bond with another male and thus was kept in a separate compartment adjacent to the non-breeder area. Keeping compartments were 5 m high and equipped with roofed areas for rain and sun-protection, several perches, live and artificial trees, bathing tubs and different types of ground substrate (sand, gravel and wood chips).

The ravens were fed twice a day with a mixture of fruits, milk products and meat. During experimental days, meat was provided only in the afternoon feeding (after the experiments). Water was provided *ad libidum*.

### Ethical note

The ravens originated from captive breeding pairs in zoos (Zoo Wels, Austria; Wildpark Haag, Austria; Nationalpark Bayrischer Wald, Germany; Gymanisium Spanga, Stockholm, Sweden). The study complied with Austrian law and local government guidelines. The study subjects remained in captivity at the Haidlhof Research Station after the completion of this study for further research.

### Experimental design and set-up

Experiments were conducted in an experimental compound that was directly connected to the birds' keeping compartments (Supplementary Fig. 1a). We used two adjacent rooms of identical size (each 3 × 4 × 4 m^3^) and equipment (sand floor; three perches; one wooden platform of 2.5 × 1 m^2^ at 3 m height; one wooden box of 1 × 1 × 1 m^3^ on the ground; one metal bowl filled with fresh water). The rooms were connected by two functional windows (35 × 25 cm^2^, 85 × 185 cm^2^), both of which were covered with wire-mesh (transparent barrier) but could also be visually closed with wooden panels (opaque barrier; Supplementary Fig. 1b). For the experiment, the left room was designated as caching room and the right room for holding potential competitors. Accordingly, individual ravens were presented with food (four pieces of cheese and dried dog food) that could be cached in the left room, whereas they did not receive any food in the right room. In the observed condition, the wooden covers on the windows were open and ravens in the right and left room could see each other. In the non-observed condition, the wooden covers were closed and the ravens in the right and left room could not see each other. Note that in both conditions, auditory contact was possible. Before the onset of the experiment, we drilled two peepholes (1 cm radius) into the wooden covers of the windows but made those non-functional by placing a small wooden board on top.

The experiment consisted of baseline trials (*n*=8) and interspersed playback trials (*n*=2). Baseline trials featured either an observed or a non-observed condition, that is, the focal subject was given food in the left compartment, whereas potential competitors were visibly present (observers) or just audibly present (non-observers) in the right compartment. Playback trials simulated the presence of conspecifics in the right compartment by playing raven sounds recorded from non-observers in the non-observed condition of the baseline phase. Note that during playback trials the covers of the door and window were closed (resembling a non-observed condition) but one of the two peepholes was open (offering possibility of being observed).

To make sure that focal subjects were knowledgeable about the open peephole, each bird got individually introduced to it from the caching room's perspective by a human experimenter before the playback trial. The human took position on the other side of peephole and got the bird's attention by calling its name and showing a piece of cheese through the peephole; when the focal bird looked through the hole, the human cached the piece of cheese in the sand and opened the door to the right room, allowing the raven to come over and search for the hidden food. Each bird had to pass the criterion of finding the cached food within 30 s two times in a row on two consecutive days before it proceeded to the playback trial. All but one bird passed the criterion in two or three sessions; one male (Rufus) appeared to be scared of the peepholes and consequently was excluded from the tests. After each individual session, the peephole was closed to prevent ravens from obtaining experience with it from the competitor's perspective.

All baseline and playback trials were administered to each ravens in a given sequence: four baseline trials (*n*=2 trials observed and *n*=2 non-observed, presented in random order) were followed by the first playback trial; another four baseline trials (*n*=2 trials observed and *n*=2 non-observed, presented in random order) were followed by the second playback trial. Irrespective of condition, a trial lasted 4 min and was followed by a 1-min feedback period, in which the large window to the competitors' room was opened and observers and non-observers, respectively, were allowed to enter the caching room and search for the caches in the presence of the focal subject. In playback trials, those birds whose sounds were played back were allowed to enter the experimental compound before the door to the caching room was opened.

### Data collection and analysis

Even though the ravens were naïve to the experimental setup, they readily cached (parts) of the food within the given time frame of 4 min. Hence, no warm-up or training phase was necessary and data collection started with the very first trials of the baseline phase (observed and non-observed, in randomized order). In case a bird ate all four food pieces within the first minute, it was given another four pieces by the experimenter and the total time increased to 5 min.

During the first baseline phase of the experiment (per bird, *n*=2 trials observed and *n*=2 non-observed, respectively), we recorded the sounds made by the competitor (non-subject) raven in the non-observed condition using a Sennheiser directional microphone (ME 66) connected to a digital sound recorder (Zoom H4n Handy Mobile 4-Track Recorder). Loud calls (‘rab') of two male and two female group members were recoded. For both sexes, the call providers were non-siblings and medium-to-high-ranking animals (second and third rank in males, top and second rank in females). Seven loud calls with high signal-to-noise ratio (0.288 s average duration) were chosen from each individual. Using Adobe Audition (Version 4.0 × 1815, 1992-2011 Adobe Systems Incorporated) peak amplitude of all calls was equalized to an average level of −9 dB Sound Pressure Level (SPL) (in Adobe Audition wave display) to prevent abrupt changes in loudness. For each focal subject, an individual acoustic stimulus set was created consisting of calls from one single non-sibling, same sex call provider. A set contained two tracks with one bout of three calls each. Within a bout, there was a call onset every 0.7 s. Both bouts in a set were composed of six different call recordings randomly assembled by a custom-made Python script (programmed by Jinook Oh). In addition, each set was assigned one of two sound recordings of a raven's wing flapping when flying up or down from the platform. Both of these recordings were peak amplitude equalized to an average level of −15 dB SPL using again Adobe Audition (same version).

During all experimental trials, the behaviour of the focal subject and its potential competitors was recorded on video, with one camera covering the right and a second camera the left experimental room. In playback trials, a loudspeaker (LD Systems Roadboy 65, frequency response: 80–15,000 Hz) was hidden behind the wooden box (1 × 1 × 1 m^3^) in the right compartment (Supplementary Fig. 1b). Sounds of a potential competitor (of the same sex as but non-affiliated to the focal bird) were played back via an iPod-touch device (fourth generation, MC540LL/A) and a radio transmitter-receiver system (Sennheiser EW 112-p G3-A Band, 516-558 MHz) three times per trial: (i) a ‘rab' call right before food was given to the focal subject for caching should alert it to the presence of a particular bird in the adjacent compartment; (ii) a wing-flapping sound presented ∼30 s after the food has been given to the focal subject should indicate that the competitor is moving and potentially interested in the focal bird's behaviour; (iii) another ‘rab' call during minute 2 should remind the focal subject that the competitor is still there. All sounds were played at natural loudness in accordance with the current wind conditions. Playback amplitude, measured at the proximate wall of the neighbouring room, reached on average 50 dBC (SPL, measured with a Voltcraft SL-100 Digital Sound Level Meter 5 Hz to 8 kHz).

Supplementary Table 2 summarizes the behavioural parameters, which were scored from the videos by Thomas Bugnyar. Only those parameters that produced significant results between the observed and non-observed condition in the initial baseline step (before the introduction of the peepholes) were used as predictors for our hypothesis (Supplementary Table 3). Note that in the peephole condition, we additionally estimated the visibility of a cache. For this purpose, a picture was taken through the peephole at the end of the experiment; all caches that were located in the area that was visible in this picture were scored as ‘inside view', whereas those that were placed in the area not visible at the picture were considered ‘outside view' (Supplementary Fig. 2). A randomly selected subsample of the videos (10%) was also coded by Stephan Reber. Inter-rater reliability calculations showed a high level of agreement for both behavioural frequencies (Cohen's Kappa: *κ*=0.785, exact *P*≤0.001) and durations (Spearman's rho correlations: *ρ*-correlation coefficients≥0.723, *P*≤0.018).

For the initial analyses, we employed generalized linear mixed models. The order of the experimental trials (first four baseline trials and playback 1, second four baseline trials and playback 2), the conditions (observed, non-observed, peephole) and the interaction between these two were used as the fixed effects (repeated measures within subjects, subjects as random effects with intercept). The target distribution was kept as a linear model, but testing was performed with robust covariances to account for potential violations of model assumptions. Initial models were stepwise reduced and the best fitting model selected with the Akaike Information Criteria. In the absence of a significant effect of trial order, we calculated the mean values per condition (non-observed, observed, peephole) for each subject and used these values for the subsequent analyses. We applied Friedman tests to compare ravens' behaviour between the observed, non-observed and peephole condition and exact Wilcoxon signed-rank tests for *post hoc* pairwise comparisons. Owing to multiple testing, we conducted Bonferroni corrections. All analyses were performed in SPSS (v. 20).

## Additional information

**How to cite this article:** Bugnyar, T. *et al.* Ravens attribute visual access to unseen competitors. *Nat. Commun.* 7:10506 doi: 10.1038/ncomms10506 (2016).

## Supplementary Material

Supplementary Information Supplementary Figures 1-2 and Supplementary Tables 1-3.

## Figures and Tables

**Figure 1:**
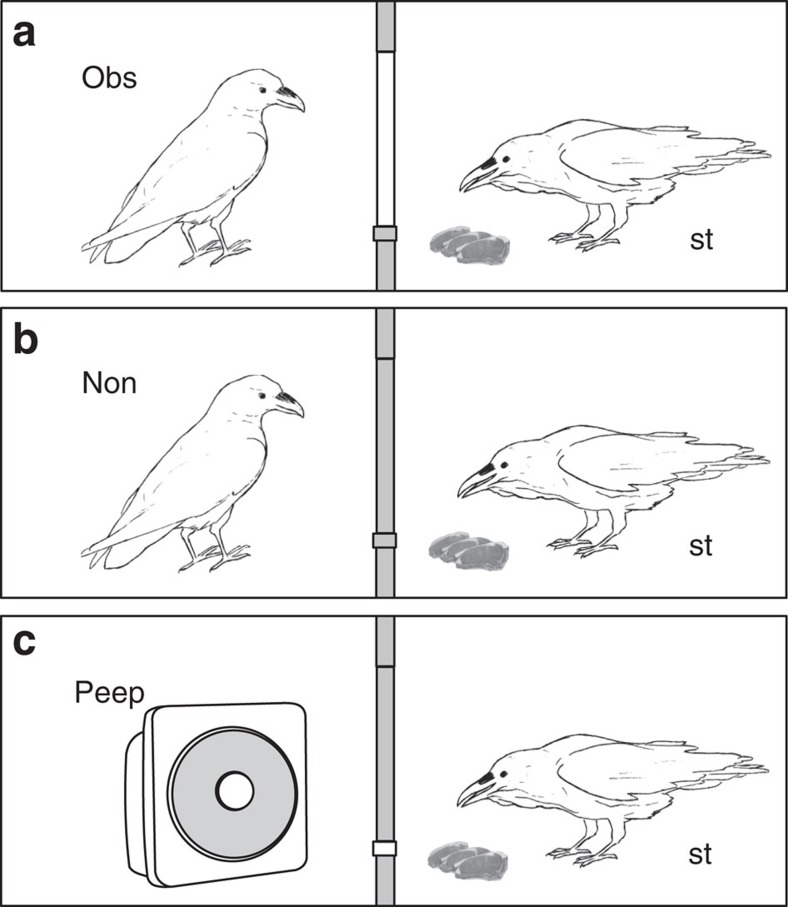
Sketch of experimental setup. (**a**) Observed (Obs) condition: The cover of the window is open (white bar) and the focal subject (storer, st) caches food in the visual presence of a conspecific (observer). (**b**) Non-observed (Non) condition: The cover of the window is closed (grey bar) and the focal subject caches food in visual isolation of a conspecific (non-observer). Both observers and non-observers make sounds in the experimental chamber, which are audible to the storer. (**c**) Peephole (Peep) condition: The cover of the window is closed (grey bar) but one of the two peepholes (small white square) is open; the focal subject caches food in the absence of any behavioural cues, whereas the presence of conspecifics is simulated via playback of sounds recorded from non-observed trials (symbolized by loudspeaker).
