# Supplementary material for: Ravens attribute visual access to unseen competitors
Source: Nat Commun. 2016 Feb 2;7:10506. doi: 10.1038/ncomms10506 (PMC4740864; doi:10.1038/ncomms10506)
Supplement: Supplementary Information — Supplementary Figures 1-2 and Supplementary Tables 1-3. [file ncomms10506-s1.pdf]

Supplementary Figure 1

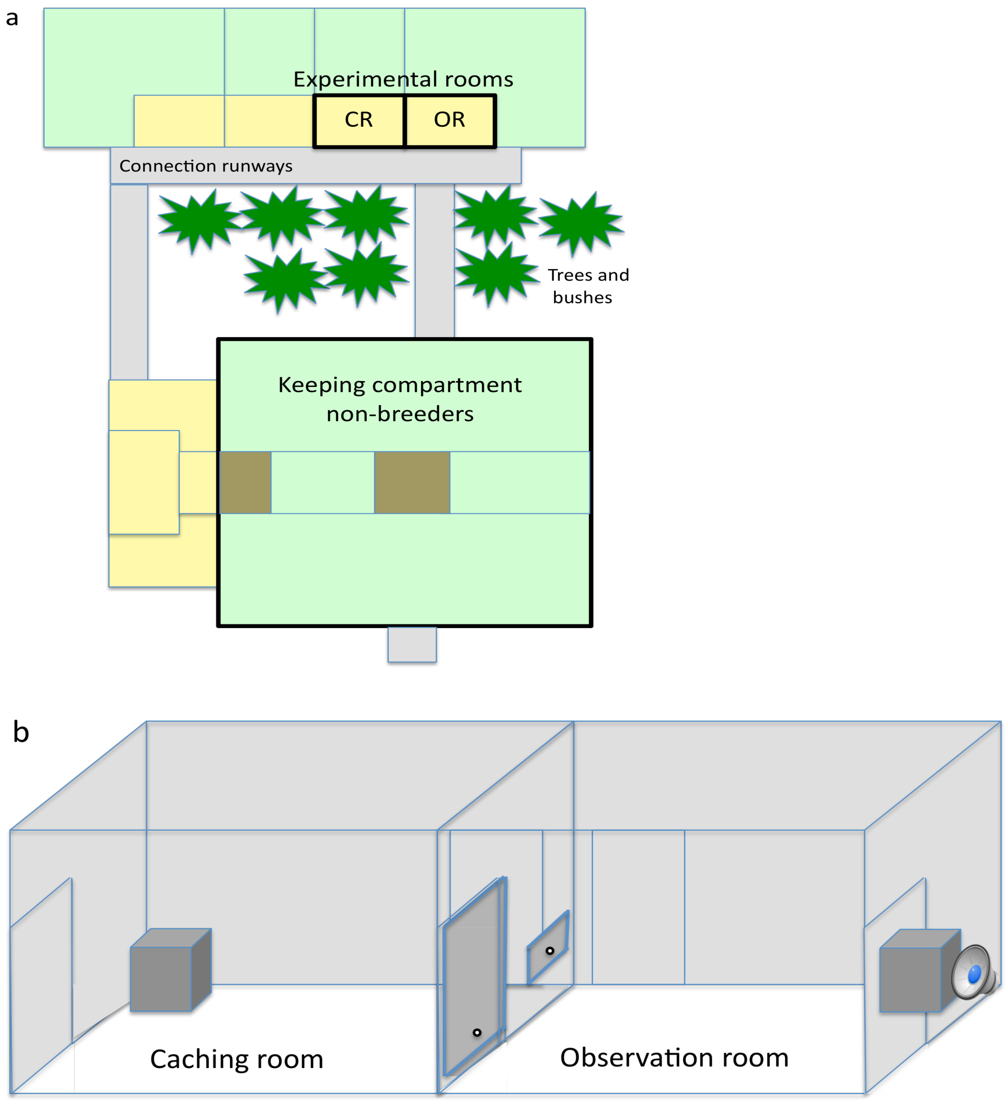

Supplementary Fig. 1: a. Sketch of aviary complex, indicating location of keeping compartments (green) and experimental rooms (yellow; CR = caching room; OR = observation room). **b.** Picture of experimental rooms, showing the two functional windows (here closed, symbolized by dark grey areas) with the peepholes (here opened, symbolized by bold framed white circles) and the location of the loudspeaker hidden behind a wooden box (dark cubicle) in the observation room.

Supplementary Figure 2

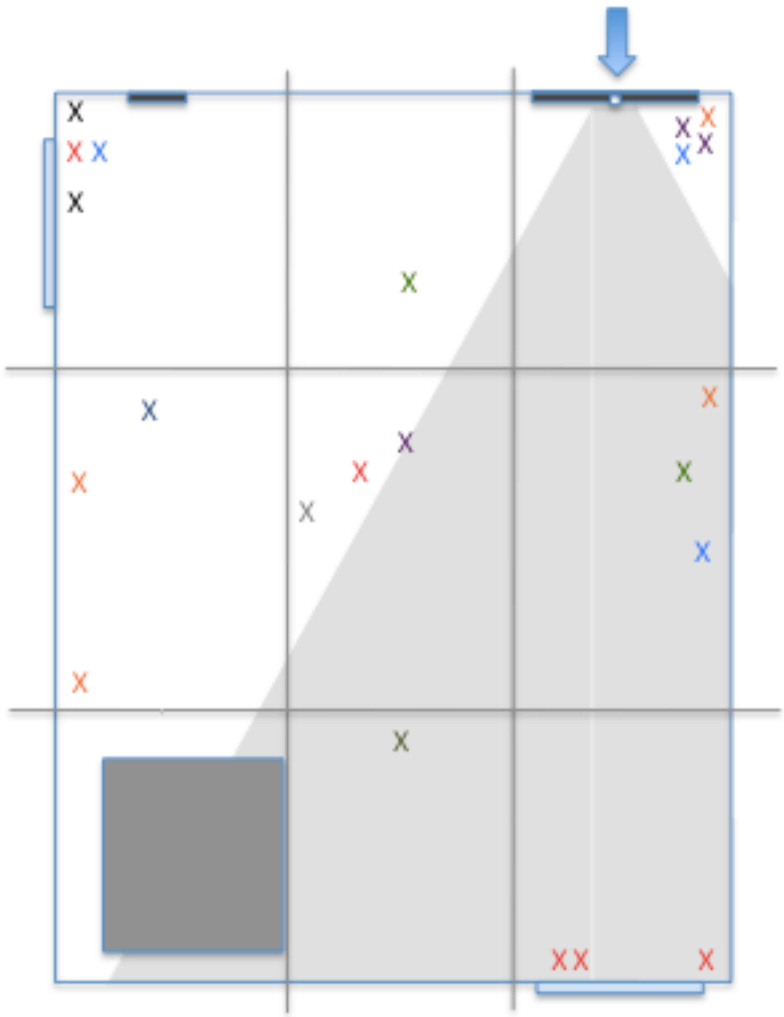

Supplementary Fig. 2: Sketch of caching room, showing the locations of caches made when the right peephole was open. Horizontal black bars on top indicate the two functional windows; arrow denotes the open peephole. Light grey area indicates the part of the room that is visible from the peephole. x denotes the location of individual caches; its colors refer to experimental subjects. The dark square on the bottom left indicates location of a wooden box. Open bars indicate doors to the keeping and experimental rooms.

Supplementary Tables:

Subject	Sex	Age	Origin	Keeping
Tom	M	2nd year	Bayrischer Wald, D	Non-breeder group
Laggie	M	2nd year	Bayrischer Wald, D	Non-breeder group
Adele	F	2nd year	Bayrischer Wald, D	Non-breeder group
George	M	2nd year	Stockholm, S	Non-breeder group
Horst	M	2nd year	Stockholm, S	Non-breeder group
Nobel	F	2nd year	Stockholm, S	Non-breeder group
Louise	F	2nd year	Stockholm, S	Non-breeder group
Paul	M	2nd year	Wels, A	Non-breeder group
Rufus	M	2nd year	Haag, A	Non-breeder group
Astrid	F	4th year	Wels, A	Pair housed

Supplementary Table 1: List of subjects participating in the study.

Parameter	Definition
Latency to cache	Time (in s) from picking up food to making the first cache
Number of caches made	Total number of caches made per trial
Number of caches recovered	Total number of caches retrieved per trial
Position of cache	Position of each cache in a horizontal grid with 3x3 units (i.e. left, middle, right; back, middle, front)
Time to finish cache	Time (in s) from placing food onto substrate/in crevice to finishing covering/leaving cache
Number of revisits with visual inspection	Total number of returns to already made caches per trial, in which the storer stands within 10cm next to the cache and visually inspects it by turning its head repeatedly to left and right
Number of revisits with improvement of cache	Total number of returns to already made caches per trial, in which the storer not only visually inspects the cache (see above) but improves it by repeatedly touching the substrate on the surface with its beak and/or placing additional cover material on top

Supplementary Table 2: Behavioral parameters measured.

Parameter	Initial baseline step<br>Non-observed	Observed
Latency to cache (s)	49±41	93±62
Time to finish a cache (s)	13±2	6±2 **
Number of caches made	1.6±0.5	1±0.5
Number of caches recovered	0.7±0.5	0.5±0.4
Number of cache revisits with visual inspection	0.23±0.26	0.17±0.27
Number of cache revisits with physical improvement	0.53±0.41	0.16±0.27 *

Supplementary Table 3: Overview of ravens' behavior in observed and non-observed condition before the introduction of the peepholes (initial baseline step; mean ± SD; Wilcoxon signed-ranks test, *=$p<0.05$, **=$p<0.01$) for all parameters measured.
